# Cranial bone thickness and density anomalies quantified from CT images can identify chronic increased intracranial pressure

**DOI:** 10.1007/s00234-024-03393-0

**Published:** 2024-06-14

**Authors:** Jiawei Liu, Jasmine Chaij, Marius George Linguraru, Brooke French, Robert Keating, Allyson L. Alexander, Antonio R. Porras

**Affiliations:** 1 Department of Biostatistics and Informatics, Colorado School of Public Health, University of Colorado Anschutz Medical Campus, Aurora, CO USA; 2 Department of Pediatric Plastic & Reconstructive Surgery, Children’s Hospital Colorado, Aurora, CO USA; 3 Sheikh Zayed Institute for Pediatric Surgical Innovation, Children’s National Hospital, Washington, DC USA; 4 Departments of Radiology and Pediatrics, George Washington University School of Medicine and Health Sciences, Washington, DC USA; 5 Department of Surgery, University of Colorado Anschutz Medical Campus School of Medicine, Aurora, CO USA; 6 Department of Neurosurgery, Children’s National Hospital, Washington, DC USA; 7 Department of Neurosurgery, University of Colorado Anschutz Medical Campus School of Medicine, Aurora, CO USA; 8 Department of Pediatric Neurosurgery, Children’s Hospital Colorado, Aurora, CO USA; 9 Departments of Pediatrics and Biomedical Informatics, School of Medicine, University of Colorado Anschutz Medical Campus, Aurora, CO USA

**Keywords:** Pediatric chronic increased intracranial pressure, Head CT image, Cranial bone anomalies

## Abstract

**Purpose:**

The diagnosis of chronic increased intracranial pressure (IIP)is often based on subjective evaluation or clinical metrics with low predictive value. We aimed to quantify cranial bone changes associated with pediatric IIP using CT images and to identify patients at risk.

**Methods:**

We retrospectively quantified local cranial bone thickness and mineral density from the CT images of children with chronic IIP and compared their statistical differences to normative children without IIP adjusting for age, sex and image resolution. Subsequently, we developed a classifier to identify IIP based on these measurements. Finally, we demonstrated our methods to explore signs of IIP in patients with non-syndromic sagittal craniosynostosis (NSSC).

**Results:**

We quantified a significant decrease of bone density in 48 patients with IIP compared to 1,018 normative subjects (*P* < .001), but no differences in bone thickness (*P* = .56 and *P* = .89 for age groups 0–2 and 2–10 years, respectively). Our classifier demonstrated 83.33% (95% CI: 69.24%, 92.03%) sensitivity and 87.13% (95% CI: 84.88%, 89.10%) specificity in identifying patients with IIP. Compared to normative subjects, 242 patients with NSSC presented significantly lower cranial bone density (*P* < .001), but no differences were found compared to patients with IIP (*P* = .57). Of patients with NSSC, 36.78% (95% CI: 30.76%, 43.22%) presented signs of IIP.

**Conclusion:**

Cranial bone changes associated with pediatric IIP can be quantified from CT images to support earlier diagnoses of IIP, and to study the presence of IIP secondary to cranial pathology such as non-syndromic sagittal craniosynostosis.

**Supplementary Information:**

The online version contains supplementary material available at 10.1007/s00234-024-03393-0.

## Introduction

The calvaria encloses the brain, cerebral spinal fluid (CSF), and cerebral blood. During childhood, it is formed by different bones separated by sutures, which allows it to accommodate space for a growing brain [1]. When pathology affects the balance between the volume inside the calvaria, and the required space for the intracranial contents, the intracranial pressure (ICP) can increase and cause symptoms such as intellectual disability, visual impairment, brain herniation, or even death [2, 3]. This imbalance may be the consequence of either growth constraint (e.g., craniosynostosis) or a buildup of excess tissue (e.g., tumors) or fluids (i.e., CSF or blood).

The identification of increased intracranial pressure (IIP) in children is challenging. Direct measurements using lumbar punctures can be inaccurate in children due to the need for anesthesia which can affect ICP in unpredictable ways. Prolonged ICP monitoring with a parenchymal sensor does provide an accurate measurement, however this requires a 24–48 h inpatient hospital stay and carries risks of hemorrhage or infection [4, 5]. Children with acute IIP caused by traumatic brain injury may present clear symptoms that prompt immediate treatment [3]. In contrast, patients with chronic IIP caused by either developmental conditions (e.g., congenital hydrocephalus, craniosynostosis) or low-grade neoplasms often show variable signs or symptoms, and diagnosis may be subjective [2, 6]. However, when signs of possible IIP are present, the patient should undergo urgent evaluation. Although papilledema has been reported to have a high specificity for the identification of IIP, its diagnostic sensitivity is variable and lower in younger patients [2, 7]. Hence, the diagnosis of chronic IIP in children is often unclear and pediatric IIP remains underdiagnosed [8, 9].This diagnostic challenge may help to explain variability in reported prevalence of IIP in children with craniosynostosis before treatment [10–15].

Radiological evaluation can provide insight into the presence of IIP in patients with chronically elevated intracranial pressure. Observations of intracranial mass lesions, hydrocephalus, sinus thrombosis, scleral flattening or optic nerve tortuosity, and compression of the dural venous sinuous can indicate IIP on intracranial magnetic resonance images (MRI)or computed tomography (CT) [16, 17]. However, these findings are variable, less common in chronic patients compared to acute cases, and may be subtle, so their identification depends on the observer’s expertise.

Previous investigations have shown that elevations in intracranial pressure cause an imbalance between bone resorption and deposition at the cranial bone plates that can result in abnormal bone thickness and density [13, 18–20]. These findings are supported by clinical observations of cranial bone thinning and density loss in patients with IIP [18, 19]. Unfortunately, since these observations are highly variable because of their dependency on age and sex, and are affected by image resolution and partial volume effects in CT images [21], they have not been routinely used to identify IIP. In this work, we hypothesize that the subtle cranial bone density and/or thickness changes as consequence of chronic IIP can be systematically quantified in children using routinely acquired CT images to support neuroradiological evaluation. Our approach leverages an age- and sex-specific normative reference of pediatric cranial bone development that represents the expected ranges of bone density and thickness at each location of the calvarium to quantify the abnormal patterns commonly observed in a dataset of children with chronic IIP of diverse etiology. Then, we present a machine learning tool that we trained to identify such quantified anomalies. Finally, we present a pilot study demonstrating the use of our methods to explore signs of IIP in an independent dataset of patients with non-syndromic sagittal craniosynostosis (NSSC) before treatment, for whom clinical reports of IIP are highly variable [8, 9, 11, 15, 22].

## Materials and methods

### Data description

Three retrospective CT image datasets of children younger than 10 years between 2005 and 2022 were obtained after approval from the internal review boards at University of Colorado Anschutz Medical Campus (protocol #20-1563) and Children’s National Hospital (protocol #3792). All CT images acquired in Dataset A, B, and C were acquired as a part of standard clinical care and hence, no CT images were acquired specifically for this retrospective study. Dataset A included CT images of 1,018 normative subjects without IIP (539 male and 479 female, age 3.08 ± 3.02 years, age range 0–10 years). These subjects were referred to the emergency room for trauma and their clinical and radiological evaluation ruled out any cranial anomaly. All these images have been used in a previous study to create a normative statistical reference of pediatric development [23] and were used in this study to create a quantitative normative reference of bone thickness and density at each location of the calvaria, accounting for the variability in the population associated with age and sex, in addition to the effects introduced by diverse image resolutions.

Dataset B contained CT images of 48 patients with diagnosis of chronic IIP (23 male and 25 female, age 4.23 ± 3.13 years, age range 0–10 years), who required surgical treatment after long-term follow-up. Strict inclusion and exclusion criteria were used to ensure an accurate diagnosis. Patients were followed clinically for at least 2 weeks with at least one clinical sign of IIP (average symptom duration 2.5 $\pm$2.9 months) in addition to at least one quantifiable sign of IIP [24, 25]. Clinical signs of IIP were defined as symptoms of headaches (*N* = 15), vision changes (*N* = 2), vomiting/nausea (*N* = 26), agitation (*N* = 7), lethargy (*N* = 9), developmental delay/loss of milestones (*N* = 4), and/or bulging/full/tense fontanelles (*N* = 3) that were consistently present during follow-up [2, 26]. Quantifiable signs of IIP included papilledema on ophthalmologic evaluation or MRI (*N* = 18), increased occipital frontal circumference (*N* = 8), variable imaging signs of IIP or hydrocephalus during neuroradiological evaluation (*N* = 44, including midline shift, ventriculomegaly, periventricular edema, and/or tortuous optic nerve), and/or direct measurement of increased intracranial pressure via intracranial pressure monitors, lumbar punctures or tense dura during surgery (*N* = 6) [24, 26–28]. See Online Resource 1 for detailed etiology of chronic IIP, clinical and quantifiable symptoms of each patient in Dataset B. Symptom presentation in all these patients was not acute and worsened progressively over the follow up period until treatment was indicated. Exclusion criteria included presentation with acute elevated intracranial pressure with symptoms such as altered mental status, respiratory failure, loss of consciousness, or signs of brain herniation. Patients were also excluded if they presented with any signs of malnutrition since it could have secondary bone effects that could bias our study. Patients with chronic IIP secondary to intracranial neoplasms were also excluded if there was bone involvement due to the possible effect of the tumor on bone density and thickness. No patients underwent any surgery, radiotherapy/chemotherapy, or any cancer treatment prior to image acquisition. For patients with chronic IIP secondary to intracranial neoplasms, only slow growing neoplasms were included (WHO Grade 1 or 2 neoplasms, and 4 WHO Grade 3 tumors consisting of ependymomas that obstructed CSF and caused ventriculomegaly). The etiology of chronic IIP in Dataset B is listed in Table 1.

Dataset C contained cross-sectional CT images of 242 patients (180 male, age 0.38 $\pm$ 0.35 years, age range 0–2 years) with diagnosis of NSSC prior to treatment. Patients older than two years in Dataset C were excluded because of insufficient data for statistical analysis in this patient group.

**Fig. 1:**
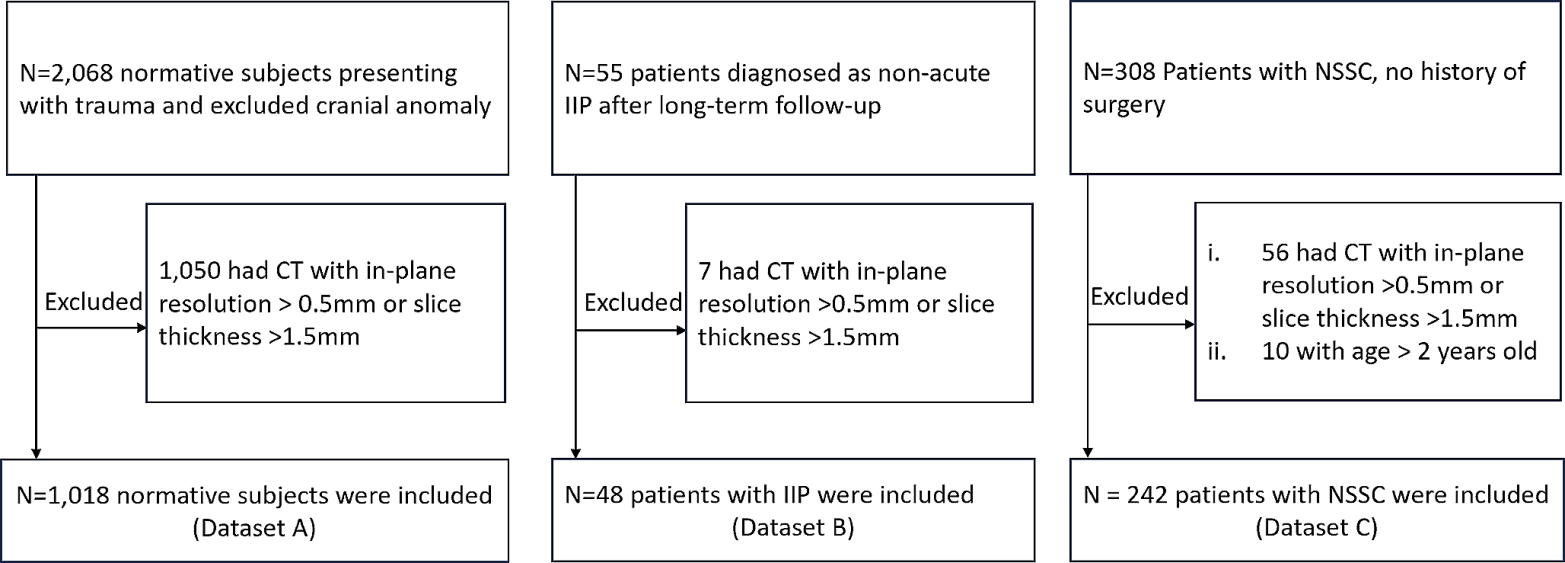
Data collection flow charts for datasets **A**, **B**, and **C**

To minimize data variability associated with image resolution and partial volume effects, images with in-plane resolution larger than 0.5 mm or slice thickness larger than 1.5 mm were excluded. In addition, patients with prior history of surgery or known genetic conditions were excluded. Table 1 presents the characteristics of the patient population, and Fig. 1 shows the data collection flow chart. The average in-plane image resolution was 0.37$\pm$0.06 mm and the slice thickness were 0.72$\pm$0.26 mm. No significant image resolution differences were found between different datasets (*P*=.88, *P*=.07 and *P* = .10 between Datasets A and B, A and C, and B and C, respectively).

**Table 1:** Details of datasets A (normative subjects), B (patients with chronic increased intracranial pressure), and C (patients with non-syndromic sagittal craniosynostosis)

Characteristic	Dataset A	Dataset B	Dataset C
**Number of patients**	1,018	48	242
**Age in years**			
Mean ± SD	3.08$\pm$ 3.02	4.23 $\pm$3.13	0.38 $\pm$ 0.35
Range	0–10	0–10	0–2
**Sex**			
Male	539 (52.95%)	23 (47.92%)	180 (74.38%)
Female	479 (47.05%)	25 (52.08%)	62 (25.62%)
**Etiology of IIP**			
Hydrocephalus	N/A	5 (10.42%)	N/A
Hemorrhage	N/A	2 (4.17%)	N/A
Idiopathic	N/A	2 (4.17%)	N/A
Arachnoid cysts	N/A	3 (6.25%)	N/A
Intracranial neoplasms	N/A	36 (75.00%)	N/A

### Standardizing the representation of the calvaria

**Fig. 2:**
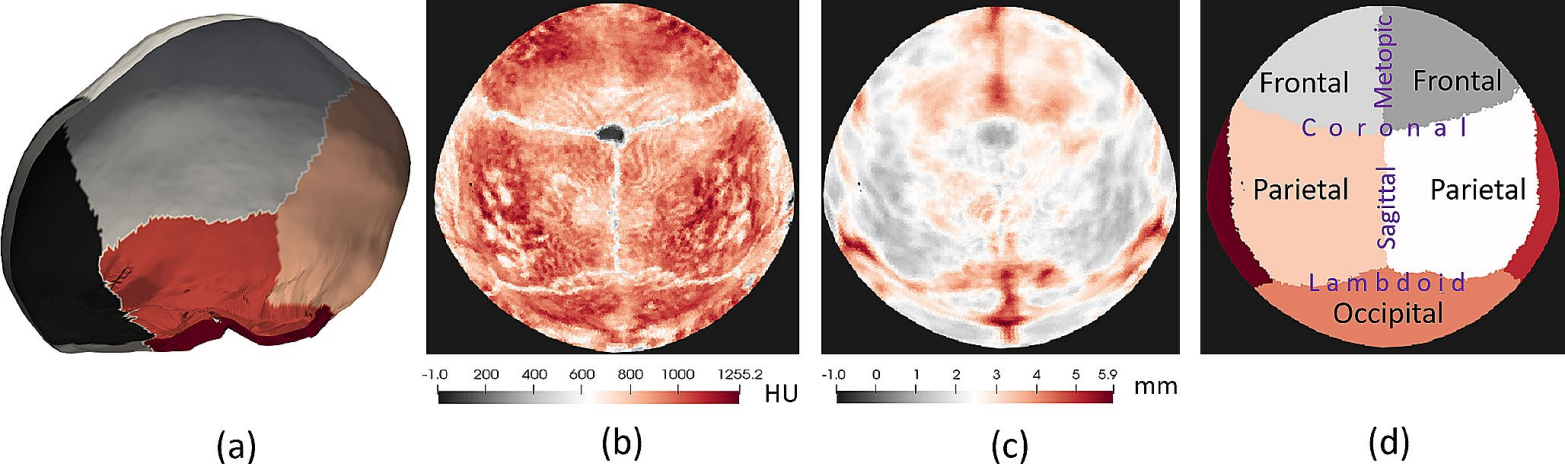
Standardized representation of the calvaria. (**a**) Cranial surface segmented from a CT image with color-coded bone labels. (**b**)-(**c**) Standardized 2-dimensional representation of the average bone density in Hounsfield units (**b**) and thickness in mm (**c**) for a 1.4-year-old normative subject. (**d**) Location of every major bone and suture

All images were resampled to a uniform in-plane resolution of 0.5 mm and slice thickness of 1.5 mm using linear interpolation to reduce data diversity associated with partial volume effects. We used publicly available state-of-the-art methods [29, 30] to automatically segment the calvaria and label the frontal, parietal, and occipital bones from the CT images (see Fig. 2). Any segmentation inaccuracies were manually corrected. After calculating cranial bone thickness (in mm) and average density (in Hounsfield units (HU)) at each location of the calvaria [30] we used spherical sampling to obtain two-dimensional representations of the calvaria. These representations were then iteratively aligned at the cranial sutures to obtain standardized anatomical representations among patients as shown in Fig. 2 (b)-(d) [30].

### Statistical analysis

#### Effect of intracranial pressure on cranial bone thickness and density

Using the standardized anatomical representations, we calculated the average cranial bone thickness and density at each cranial bone and suture for every patient. For improved interpretability, we then used linear regression to compare statistically the distributions observed in the normative population (Dataset A) and in the patients with chronic IIP (Dataset B). Since cranial growth rates in the first two years are substantially faster than between 2 and 10 years [31], we divided our datasets into these two age groups. Then, we used linear regression to quantify the effect of IIP on cranial bone thickness and density accounting for age and sex. Since image resolution can affect the quantification of thickness and density in CT images [32, 33], we also accounted for the original CT image voxel volume using the expression:

1 $${Y_i} = {\beta _0} + {\beta _1}*Ag{e_i} + {\beta _2}*{V_i} + {\beta _3}{\rm{*}}Se{x_i} + {\beta _4}*II{P_i}{\rm{\;\;}}$$

where ${Y}_{i}$ is the local cranial bone thickness or density of patient $i$ at a cranial location, ${\beta }_{j}, j=\{0,\cdots ,4\}$ are the regression parameters, $Ag{e}_{i}$ is the age of patient $i$, ${V}_{i}$ the original image voxel volume in $m{m}^{3}$, and $Se{x}_{i}$ and $II{P}_{i}$ are binary indicators representing if patient $i$ is male or presents IIP, respectively. The significance of the effect of IIP was evaluated using t-tests.

#### Automatic identification of IIP

After quantifying the significance of the differences in cranial bone thickness and density between normative subjects and patients with chronic IIP, we built a machine learning-based method to automatically identify patterns associated with chronic IIP. First, we created statistical normative reference models of bone thickness and density using Dataset A at each region of the calvaria using a similar formulation to other works [23] but also accounting for image resolution. Specifically, we constructed local non-linear regression models from birth to 10 years as:

2 $$\eqalign{& {Y_i} = {\alpha _0} + {\alpha _1}*Ag{e_i} + {\alpha _2}{\rm{*}}Se{x_i} \cr & + {\alpha _3}*{\rm{arcsinh}}\left( {{\alpha _4}*Ag{e_i}} \right) + \cr & {\alpha _5}*Ag{e_i}{\rm{*}}Se{x_i} + {\alpha _6}*{V_i} \cr}$$

where ${\alpha }_{j},j=\{0,\cdots ,6\}$ are the regression parameters. Model parameters were estimated using the Gauss-Newton algorithm [34] with the goal of minimizing the squared difference between the real observations in Dataset A and our model estimations.

Then, for every subject in Datasets A and B, we calculated their thickness and density anomalies as the distance between the quantified values from their CT images and the regressed normative reference values using Eq. (2). For symmetric structures (i.e., the two frontal and two parietal bones, and the two coronal and lambdoid sutures), we calculated the average and absolute difference between the left and right structures. Principal component analysis was then used to represent the quantified information to eliminate collinearities. After discarding the principal components with zero variance, we trained a logistic regression classifier to distinguish between patients with and without chronic IIP, after using recursive feature elimination [35] for feature selection. During classifier training, we used a weighting scheme [36] to compensate for class imbalance between Datasets A (*n* = 1,018) and B (*n* = 48) so both groups had equal contribution to the classification model. Evaluation was performed using leave-one-out cross-validation.

#### Study of signs of IIP in patients with NSSC

Following the approach presented in Sect. 2.3.1, we quantified the statistical significance of the differences in bone thickness and density between patients with NSSC (Dataset C), the normative population (Dataset A) and patients with IIP (Dataset B) by modifying Eq. (1) to account for NSSC as:

3 $$\eqalign{& {Y_i} = {\eta _0} + {\eta _1}*Ag{e_i} + {\eta _2}* \cr & Se{x_i} + {\eta _3}*{V_i} + {\eta _4}*II{P_i} \cr & + {\eta _5}*NSS{C_i} \cr}$$

where ${\eta }_{j},j=\{0,\cdots ,5\}$ are the regression parameters, and $NSS{C}_{i}$ is a binary indicator representing if patient $i$ is diagnosed with NSSC. Note that $NSS{C}_{i}$ and ${IIP}_{i}$ are mutually exclusive.

We then studied if patients with NSSC present signs of chronic IIP using a similar classifier to the one trained in Sect. 2.3.2 to identify chronic IIP. Specifically, after calculating thickness and density anomalies, we discarded the information that was uniquely characteristic of the pathology of patients in Dataset C with NSSC (i.e., suture information and bone thickness as presented in the results of Sect. 3.3) to avoid introducing quantitative biases exclusive of NSSC and enable model generalization. Then, we retrained our classifier to classify patients with IIP (Dataset B) from normative subjects (Dataset A). After training, we used our model to predict the risk of IIP in the independent dataset of patients with NSSC (Dataset C).

## Results

### Effect of intracranial pressure on cranial bone thickness and density

**Fig. 3:**
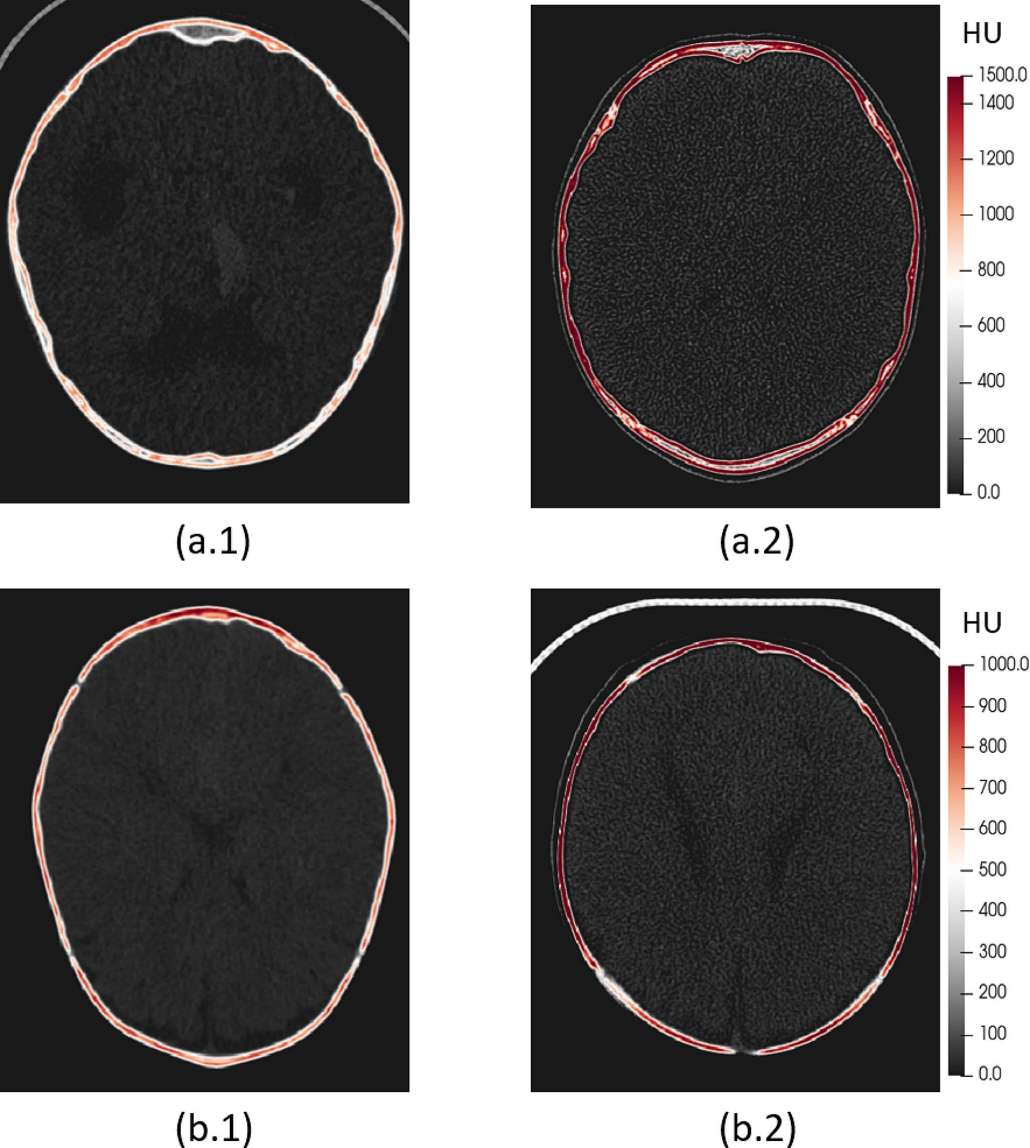
Qualitative demonstration of the bone anomalies in the axial CT image slices of patients with chronic IIP (**a.1**) and NSSC (**b.1**), compared to age- and sex-matched normative subjects (**a.2** and **b.2**). (**a.1**) 7.7-year-old male patient with chronic IIP secondary to intracranial neoplasms and decreased calvarial bone density. (**a.2**) 7.7-year-old normative male subject. (**b.1**) 10.9-month-old female patient with untreated NSSC (**b.1**) and decreased calvarial bone density. (**b.2**) 10.9-month-old female normative reference (**b.2**). Cranial bone density in Hounsfield Units (HU) is represented color-coded

Table 2 presents the differences in cranial bone thickness and density between normative subjects and patients with IIP. A p-value threshold of 0.05 was used in this study to determine statistical significance. No significant bone thickness differences were found at the cranial bone plates except for the occipital bone under 2 years of age. However, significant bone thinning was found in patients with IIP around the open cranial sutures (except for the normally fused metopic suture after 2 years of age). A general significant decrease of density at the cranial bones was observed in patients with IIP at all ages (*p* < .001). While significant decrease (*p* < .001) in bone density was also found in all sutures between birth and 2 years, differences compared to normative were only significant at the coronal and the lambdoid sutures between 2 and 10 years. In Fig. 3, we present a representative example of the cranial bone anomalies in a patient with chronic IIP from Dataset B (a.1) compared to an age- and sex-matched normative reference from Dataset A (a.2).

**Table 2:** Mean differences in local cranial bone thickness and bone density between patients with IIP (Dataset B) and normative subjects (Dataset A)

	Difference in thickness (mm)			Difference in bone density (HU)		
Age group (years)	Difference	SD	p-value	Difference	SD	p-value
**Frontal bones**						
0–2	0.08	0.10	0.42	-175.96	29.45	< 0.001**
2–10	0.13	0.12	0.27	-128.00	25.04	< 0.001**
**Parietal bones**						
0–2	-0.03	0.08	0.68	-176.06	26.25	< 0.001**
2–10	-0.13	0.11	0.24	-97.43	21.91	< 0.001**
**Occipital bone**						
0–2	0.20	0.09	0.03*	-124.84	24.96	< 0.001**
2–10	0.19	0.11	0.10	-177.34	20.83	< 0.001**
**Bone average**						
0–2	0.05	0.08	0.56	-165.42	25.40	< 0.001**
2–10	0.02	0.11	0.89	-121.16	20.96	< 0.001**
**Metopic suture**						
0–2	-0.33	0.11	0.004*	-144.22	30.72	< 0.001**
2–10	0.11	0.15	0.47	-9.73	26.28	0.71
**Coronal sutures**						
0–2	-0.53	0.08	< 0.001**	-236.32	18.87	< 0.001**
2–10	-0.32	0.12	0.006*	-163.25	19.33	< 0.001**
**Sagittal suture**						
0–2	-0.42	0.07	< 0.001**	-207.99	21.61	< 0.001**
2–10	-0.36	0.11	0.001*	-22.21	23.40	0.34
**Lambdoid sutures**						
0–2	-0.35	0.09	< 0.001**	-188.55	24.05	< 0.001**
2–10	-0.25	0.13	0.05	-88.13	20.93	< 0.001**

Patients are grouped by age into categories of 0–2 years and 2–10 years. SD represents standard deviation. * and ** represent significant differences for *P* < .05 and *P* < .001, respectively, evaluated using t-tests. Positive differences indicate higher values in patients with IIP, and negative differences indicate lower values in patients with IIP

### Automatic identification of IIP

**Fig. 4:**
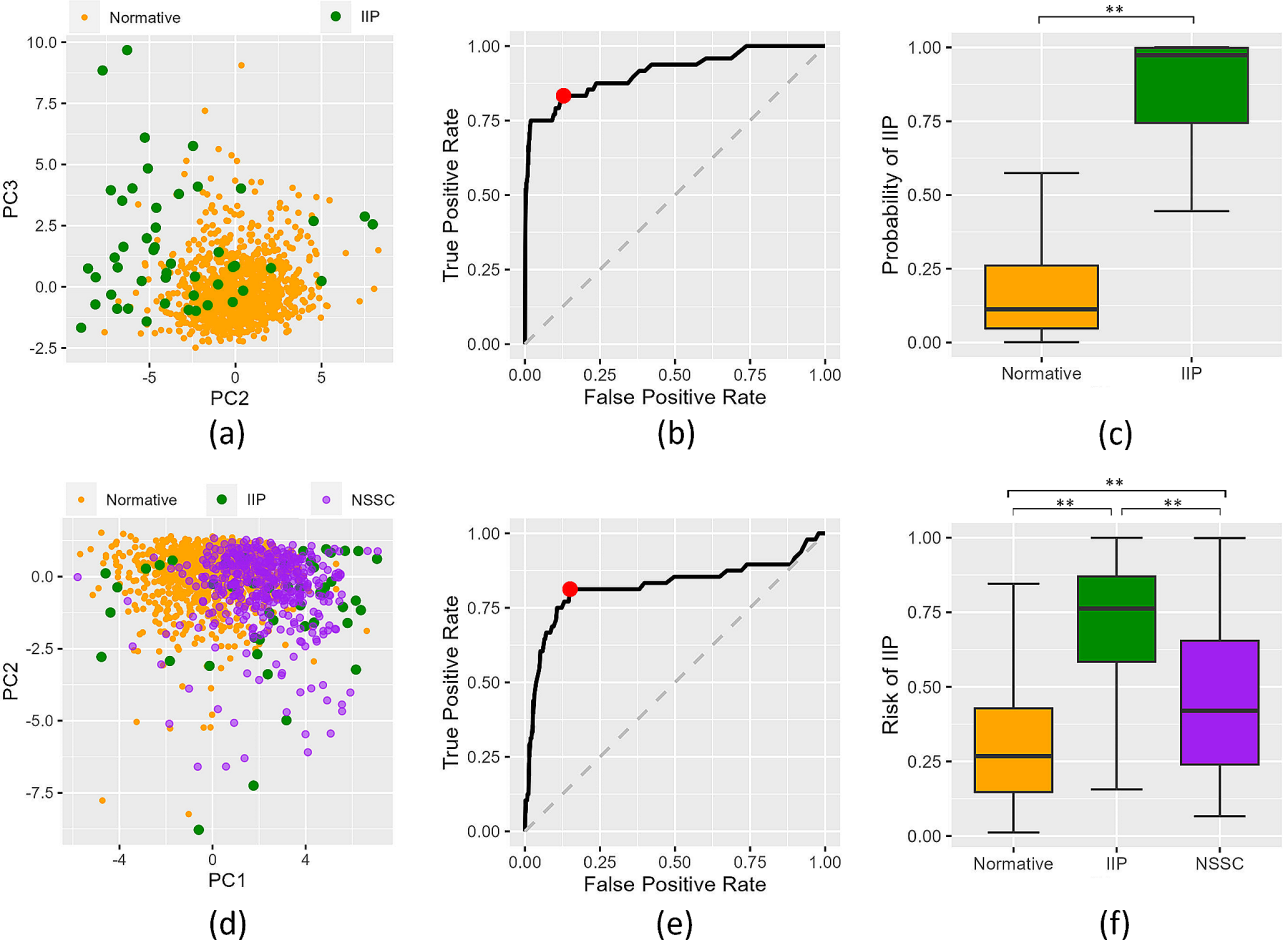
(**a**)-(**c**): Results from the classifier trained using cranial bone thickness and density anomalies to identify IIP (Sect. 2.4 and 3.2). (**a**) First two selected principal components with highest variance for the normative subjects (orange circles) and patients with IIP (green circles), respectively. (**b**) Receiver operating characteristic (ROC) curve. The probability threshold that maximizes Youden’s index is represented in red. (**c**). Box plot representing the cross-validated risk of IIP in the normative subjects and patients with IIP, evaluated using leave-one-out cross-validation. ** represents significant differences for *P* < .001, evaluated using Mann Whitney U-Test. (**d**)-(**f**): Results from the classifier trained to identify IIP using only bone density anomalies at the bone plates and tested to identify signs of IIP in patients with NSSC (Sect. 2.5 and 3.3). (**d**). First two selected principal components with highest variance for the normative subjects (orange circles), patients with IIP (green circles), and patients with NSSC (purple circles), respectively. (**e**) ROC curve. (**f**) Box plot representing the probability of IIP in the normative subjects, patients with IIP, and patients with NSSC, respectively. The probabilistic risk of IIP for the normative subjects and patients with IIP were quantified using cross-validation. The probabilistic risk of IIP for patients with NSSC were quantified using the final model trained with all normative subjects and patients with IIP

After feature selection via recursive feature elimination, six principal components with non-zero variance were selected to train our logistic regression classifier. Figure 4 (a) shows our data represented using the first two selected principal components with the highest variance. Our leave-one-out cross-validation results yielded 86.96% (95% CI: 84.76%, 88.89%) accuracy, 87.13% (95% CI: 84.88%, 89.09%) specificity, and 83.33% (95% CI: 69.24%, 92.03%) sensitivity. Among the eight misclassified patients with IIP (16.67% of the total), one had congenital hydrocephalus (20.00% of this patient group), two had idiopathic intracranial hypertension (100.00% of this patient group), one had arachnoid cysts (33.33% of this patient group), and four had intracranial neoplasms (11.11% of this patient group). No patients with hemorrhage were misclassified. Figure 4. (b) shows the Receiving Operator Characteristics (ROC) curve with an area (AUROC) of 0.91. The distribution of probabilities calculated during cross-validation are represented in Fig. 4 (c). We obtained a significantly higher average cross-validation risk of IIP (19.08 $\pm$ 20.29%) in the normative subjects than in patients with IIP (78.44 $\pm$ 32.31%, *P*<.001 with Mann Whitney U-Test).

### Study of signs of IIP in patients with NSSC

Table 3 presents the differences in local cranial bone thickness and density of our patients with NSSC (Dataset C) compared to the normative subjects (Dataset A) and patients with chronic IIP (Dataset B). Patients with NSSC exhibited significant cranial thickening compared to both normative subjects and patients with IIP (*p* < .001). In addition, patients with NSSC generally presented a significant bone density decrease compared to the normative subjects at the cranial bones (*p* < .001), which was not different to the decreases observed in patients with chronic IIP. We also found a significant increase of bone density at the sutures in patients with NSSC compared to patients with IIP (*p* < .001). When compared to normative subjects, the bone density of patients with NSSC was not different in the metopic suture, was higher at the sagittal suture (because of fusion) and was lower at the coronal and lambdoid sutures. Figure 3 (b.1) and (b.2) show a qualitative example.

**Table 3:** Mean differences in local cranial bone thickness and bone density of patients with NSSC compared to patients with IIP and normative subjects

	Difference in thickness (mm)			Difference in bone density (HU)		
Patient group	Difference	SD	p-value	Difference	SD	p-value
**Frontal bones**						
NSSC vs. Normative	0.47	0.03	< 0.001**	-141.30	9.82	< 0.001**
NSSC vs. IIP	0.41	0.10	< 0.001**	33.41	28.72	0.25
**Parietal bones**						
NSSC vs. Normative	0.38	0.03	< 0.001**	-166.31	8.75	< 0.001**
NSSC vs. IIP	0.41	0.08	< 0.001**	9.65	25.60	0.71
**Occipital bone**						
NSSC vs. Normative	0.36	0.03	< 0.001**	-122.26	8.87	< 0.001**
NSSC vs. IIP	0.17	0.09	0.06	-1.56	25.92	0.95
**Bone average**						
NSSC vs. Normative	0.40	0.03	< 0.001**	-150.29	8.47	< 0.001**
NSSC vs. IIP	0.36	0.08	< 0.001**	13.95	24.78	0.57
**Metopic suture**						
NSSC vs. Normative	0.29	0.04	0.001*	-6.50	10.69	0.54
NSSC vs. IIP	0.64	0.11	< 0.001**	140.18	31.26	< 0.001**
**Coronal sutures**						
NSSC vs. Normative	0.31	0.03	< 0.001**	-74.17	6.39	< 0.001**
NSSC vs. IIP	0.84	0.08	< 0.001**	163.78	18.69	< 0.001**
**Sagittal suture**						
NSSC vs. Normative	0.62	0.03	< 0.001**	160.77	8.39	< 0.001**
NSSC vs. IIP	1.04	0.08	< 0.001**	368.06	24.53	< 0.001**
**Lambdoid sutures**						
NSSC vs. Normative	0.17	0.03	< 0.001**	-133.38	7.88	< 0.001**
NSSC vs. IIP	0.54	0.09	< 0.001**	56.62	23.04	0.01*

All patients were younger than 2 years. SD represents standard deviation. * and ** represent significant differences for *P* < .05 and *P* < .001, respectively, evaluated using t-tests. Positive differences indicate higher values in patients with NSSC, and negative differences indicate lower values in patients with NSSC

After retraining our classifier to identify patients with IIP from normative subjects as described in Sect. 2.3.3, five principal components with non-zero variance were selected using recursive feature elimination. Figure 4 (d) shows the first two selected principal components. Figure 4 (e) shows the ROC curve, with an AUROC of 0.82. The leave-one-out cross-validation yielded 84.80% (95% CI: 82.47%, 86.88%) accuracy, 84.97% specificity (95% CI: 82.59%, 87.08%), and 81.25% (95% CI: 66.90%, 90.56%) sensitivity, which are not statistically different to the classification results obtained when using thickness and suture information (*P* = .11 with McNemar’s test). Figure 4. (f) shows the distribution of the risk of IIP in normative subjects, patients with chronic IIP and patients with NSSC. The classifier quantified a significantly higher risk of IIP in patients with NSSC (45.79 $\pm$ 25.42%) than in normative subjects (30.83 $\pm$ 20.54%, *P*<.001 with Mann Whitney U-Test), and lower than in patients with confirmed chronic IIP (66.70% $\pm$ 28.37%, *P*<.001). 36.78% (95% CI: 30.76%, 43.22%) of our patients with NSSC were identified as having high risk of IIP.

## Discussion

We studied cranial bone anomalies in patients with chronic IIP caused by congenital anomalies or low-grade neoplasms requiring longitudinal evaluation until confirmation of elevated pressure. We showed quantitatively that these children present a significant decrease in calvarial bone density compared to normative subjects. Although this decrease may be more subtle than in patients with acute IIP and may be missed by the human eye during neuroradiological evaluation, we showed that it can be accurately and systematically identified from routinely acquired CT images using quantitative tools. Importantly, our methods account for the variability associated with patient age, patient sex, and diverse image resolutions.

The cranial sutures enable outward cranial bone growth during development as the meninges expand to compensate for the pressure increase from a growing brain. Osteocytes are mechanosensitive and respond to local increases in intracranial pressure, which allows the cranial bones to remodel through the coordinated action of osteoblasts and osteoclasts to form and absorb bone, respectively [37]. This coordinated system helps maintain the balance between bone resorption at the inner calvarial layer, deposition at the sutures and outer calvarial layer, and remodeling [31]. Given the subtlety and variability of cranial bone anomalies in patients with chronic IIP [2, 38], the lack of age- and sex-specific normative references of cranial bone thickness and density, and the unavailability of quantitative tools to enable systematic studies, reports of cranial bone anomalies in children with chronic IIP are variable and contradictory [2, 22]. Hence, longitudinal or invasive clinical evaluation is often needed before a diagnosis can be made. Our study is the first showing that, despite their potential subtleties and variability, local cranial bone changes associated with chronic IIP can be systematically quantified to support neuroradiological evaluation.

Interestingly and unlike other reports [18, 19], we did not find significant thinning at the cranial bone plates, which explains the difficulties of pediatric neuroradiologists identifying these anomalies. While previous studies reported significant calvaria thinning, there were no significant thickness differences in the zygoma. Additionally, the differences found in calvarial thickness in those studies were not significant for a significance level of 0.01 [18, 19]. We hypothesize that patient age is the main reason why cranial bone thinning was not found in our study. First, it has been shown that pediatric patients retain the ability to re-ossify calvarial defects during early development [31] unlike adults. Moreover, our patients presented with a non-acute and chronic intracranial pressure increase, previous studies show that children can effectively compensate for a potential bone thinning associated with an intracranial pressure increase until a threshold beyond which cranial bone thinning can be observed [31, 37]. Hence, bone density loss may be the only bone anomaly that can systematically be quantified in patients with a non-acute intracranial pressure elevation. Another possible contributing factor may be an insufficient image resolution to find significant bone thickness anomalies in patients with chronic IIP. We only observed significant bone thinning around the open cranial sutures (i.e., no differences were found in the metopic suture area after 2 years). This may be caused by the increased pressure inducing larger bone plate separation at the sutures as the calvarial bones continue to expand outwards. This explanation aligns with the increased intracranial volumes observed in pediatric patients with IIP [14].

We evaluated if patients with chronic IIP could be identified automatically using only bone thickness and density observations from CT images. Our cross-validation results demonstrated 83.33% sensitivity and 87.13% specificity, in addition to estimating a significantly higher risk for patients with chronic IIP compared to normative. These results show the potential of our approach to assist during pediatric neuroradiological evaluation.

We also demonstrated the use of our methods to study signs of IIP in patients with NSSC, for whom reports of IIP are highly variable [8, 9, 11, 15, 22]. These patients presented a generalized density loss at the cranial bones compared to normative subjects that was not different from patients with IIP, suggesting a pressure increase. In the sutures, lower bone density compared to normative was only observed in those in which bone growth occurs perpendicular to the fused sagittal suture (i.e., coronal and lambdoid sutures). This is related to a higher pressure in the direction parallel to the fused sagittal suture, which is also responsible for the compensatory anteroposterior overdevelopment that causes scaphocephaly in these patients [39].

We found a generalized significant bone thickening in patients with NSSC compared to normative subjects and patients with chronic IIP. This interesting finding may be related to our patients with NSSC presenting less pressure increase than our patients with chronic IIP, since they usually undergo early surgical treatment before presenting clear symptoms of IIP. Previous studies [19, 21] have suggested a threshold of pressure increase under which the imbalance in the osteoblastic and osteoclastic action results in cranial bone thickening, which aligns with our findings. In addition, since the causes of craniosynostosis are unknown in these patients, a genetic disorder causing their pathology may also disrupt the normal osteogenesis processes affecting bone thickness. Although our study with a large dataset shows a significant abnormal pattern in this patient population compared to the normative, further studies are needed to confirm the specific causes for these observations.

Although identifying IIP is a significant deciding factor in the timing of surgical treatment for craniosynostosis and other developmental pathologies affecting the cranium-brain complex [2, 10–12], IIP is underdiagnosed [11, 15] and often presents with inconclusive neuroradiological evaluation [38, 40]. Our methods provide a quantitative framework to identify patients at risk of IIP using routinely acquired CT images. We identified that 36.78% of patients with NSSC present signs of IIP, which is within the reported range of prevalence of IIP in patients with non-syndromic craniosynostosis (range from 15–20% [11, 15, 22] to 44% in young patients [9], 69.4% in older untreated patients [8], and 30–40% in patients with syndromic craniosynostosis [10, 11, 15]). Since direct intracranial pressure measurements using monitors or lumbar punctures are often avoided due to the invasive nature of these procedures, future clinical validation in this patient group may be challenging. A possible pathway to seek additional validation of our approach could include a correlation study between the estimated risk of IIP and the phenotypic severity of their malformations [10, 15], which are a direct consequence of the compensatory overdevelopment caused by the increased intracranial pressure [41].

One limitation of this study is related to the limited CT image resolution and partial volume effects. Although our strict exclusion criteria aimed to minimize data variability, there were no significant resolution differences between our datasets, and our pre-processing steps and models accounted for image resolution, partial volume effects may still cause inaccuracies in our quantifications [32, 33]. Another limitation is that Dataset B is comprised of primarily patients with intracranial neoplasms, which may influence our model’s ability to generalize to other causes of IIP. Hence, we are working in building collaborations to increase patient diversity in Dataset B to achieve higher performance and generalizability of our methods. We expect to validate our model in patients with chronic IIP secondary to other non-syndromic or syndromic craniosynostosis in future studies. Importantly, a prospective clinical study on a larger population group needs to be considered to clinically validate our methods.

## Conclusion

In this work, we have shown that quantitative statistical and machine learning methods can systematically identify local cranial bone thickness and density anomalies that are caused by chronic IIP in routinely acquired pediatric CT images. In addition, we have demonstrated the application of our methods to explore signs of elevated intracranial pressure in patients with NSSC. Our results show the potential of our methods to support pediatric neuroradiological assessment in achieving earlier noninvasive diagnoses of IIP without long-term follow-up. Our methods are also highly valuable to investigate elevated intracranial pressure secondary to cranial pathology.

## Electronic supplementary material

Below is the link to the electronic supplementary material.

Supplementary Material 1

## Data Availability

Head CT images of pediatric patients used in this study cannot be made public due to HIPAA regulations. The methods and model is available in https://github.com/cuMIP/BoneSignsOfIIP.

## Abbreviations

IIP: increased intracranial pressure
NSSC: non-syndromic sagittal craniosynostosis
CSF: cerebral spinal fluid
