# Supplementary material for: Cranial bone thickness and density anomalies quantified from CT images can identify chronic increased intracranial pressure
Source: Neuroradiology. 2024 Jun 14;66(10):1817–28. doi: 10.1007/s00234-024-03393-0 (PMC11424726; doi:10.1007/s00234-024-03393-0)
Supplement: Supplementary file 1 — Supplementary Material 1 [file 234_2024_3393_MOESM1_ESM.docx]

**Title**: Cranial bone thickness and density anomalies quantified from pediatric CT images can identify chronic increased intracranial pressure

# Appendix

| Table 1. Etiology, clinical signs, and quantifiable signs of patients with chronic increased intracranial pressure (IIP) in Dataset B. | | | |
| --- | --- | --- | --- |
| Patient ID | Etiology of IIP | Clinical signs | Quantifiable signs |
| 1 | Ruptured Arachnoid Cyst | Headaches | Midline shift on CT; Papilledema |
| 2 | Tumor | Headaches; Lethargy; Vomiting | Tortuous optic nerve on MRI;  Papilledema |
| 3 | Tumor | Headaches | Papilledema |
| 4 | Tumor | Headaches | Papilledema |
| 5 | Tumor | Headaches | Papilledema |
| 6 | Idiopathic Hydrocephalus | Headaches; Vomiting | Papilledema |
| 7 | Tumor | Agitation; Lethargy | Ventriculomegaly on CT; Tense dura during surgery after CT image acquisition |
| 8 | Tumor | Vision changes | Periventricular edema on CT; Papilledema |
| 9 | Tumor | Vision changes | Tortuous optic nerves and papilledema on MRI |
| 10 | Tumor | Headaches; Vomiting | Periventricular edema on CT |
| 11 | Idiopathic Hydrocephalus | Headaches | Increased intracranial pressure on serial lumbar punctures; Papilledema |
| 12 | Tumor | Vomiting | Marked ventriculomegaly with trans-ependymal edema on CT; Increased occipitofrontal circumference |
| 13 | Tumor | Lethargy; Vomiting | Ventriculomegaly CT; Tortuous optic nerve on MRI; Papilledema |
| 14 | Tumor | Agitation; Loss of milestones | Ventriculomegaly on CT |
| 15 | Tumor | Agitation; Lethargy; Vomiting | Trans-ependymal edema on CT |
| 16 | Hemorrhage | Agitation; Bulging fontanelle | Intracranial pressure monitor showed increased intracranial pressure |
| 17 | Tumor | Vomiting | Papilledema; Increased occipitofrontal circumference |
| 18 | Tumor | Headaches; Vomiting | Periventricular edema on CT |
| 19 | Tumor | Headaches; Vomiting | Periventricular edema on CT; Papilledema |
| 20 | Tumor | Vomiting | Ventriculomegaly on CT; Increased occipitofrontal circumference |
| 21 | Congenital Hydrocephalus | Full fontanelles; Lethargy | Periventricular edema on CT; Increasing occipitofrontal circumference |
| 22 | Tumor | Vomiting | Edema of optic nerve sheath and ventriculomegaly on MRI |
| 23 | Tumor | Headaches; Vomiting | Periventricular edema on CT; papilledema, |
| 24 | Arachnoid Cyst | Headaches | Midline shift on CT; Papilledema |
| 25 | Tumor | Developmental delay; Vomiting | Ventriculomegaly on CT |
| 26 | Tumor | Lethargy; Vomiting | Periventricular on CT; Papilledema |
| 27 | Tumor | Vomiting | Edema on optic nerve sheath and ventriculomegaly on MRI |
| 28 | Congenital Hydrocephalus | Headaches; Vomiting | Periventricular edema on CT |
| 29 | Hemorrhage | Agitation | Ventriculomegaly on CT;  Increasing occipitofrontal circumference |
| 30 | Tumor | Headaches; Vomiting | Periventricular edema on MRI; Papilledema |
| 31 | Tumor | Vomiting | Mass effect on MRI; Ventriculomegaly on CT; Tense dura during surgery done after CT acquisition |
| 32 | Tumor | Vomiting | Periventricular edema on CT; Dilated optic nerve sheaths on MRI |
| 33 | Tumor | Vomiting | Periventricular edema on CT; Opening pressure very elevated on lumbar puncture |
| 34 | Tumor | Agitation; Lethargy; Vomiting | Periventricular edema on CT |
| 35 | Congenital Hydrocephalus | Agitation | Periventricular edema on CT; Increasing occipitofrontal circumference |
| 36 | Tumor | Nausea; Vomiting | Periventricular edema on CT |
| 37 | Tumor | Vomiting | Periventricular edema on CT |
| 38 | Arachnoid Cyst | Lethargy; Bulging /tense fontanelle | Ventriculomegaly and midline shift on CT |
| 39 | Congenital Hydrocephalus | Developmental delay | Ventriculomegaly on CT; Massively increased occipitofrontal circumference; Increased pressure noted during operation done after CT acquisition |
| 40 | Congenital Hydrocephalus | Developmental delay | Periventricular edema on CT; Macrocephaly |
| 41 | Tumor | Headaches; Vomiting | Trans-ependymal edema on CT; Papilledema |
| 42 | Tumor | Headaches; Vomiting | Trans-ependymal edema on CT; Papilledema |
| 43 | Tumor | Vomiting | Trans-ependymal edema on CT |
| 44 | Tumor | Headaches; Vomiting | Periventricular edema on CT; Papilledema |
| 45 | Tumor | Headaches; Nausea; Vomiting | Periventricular edema on CT |
| 46 | Tumor | Vomiting | Periventricular edema on CT; Increased occipitofrontal circumference |
| 47 | Tumor | Headaches; Lethargy Vomiting | Periventricular edema on CT |
| 48 | Tumor | Headaches; Vomiting | Periventricular edema on CT |

|  |
| --- |
